# Multimer Analysis of Von Willebrand Factor in Von Willebrand Disease with a Hydrasys Semi-Automatic Analyzer—Single-Center Experience

**DOI:** 10.3390/diagnostics11112153

**Published:** 2021-11-20

**Authors:** Ingrid Skornova, Tomas Simurda, Jan Stasko, Jana Zolkova, Juraj Sokol, Pavol Holly, Miroslava Dobrotova, Ivana Plamenova, Jan Hudecek, Monika Brunclikova, Alena Stryckova, Peter Kubisz

**Affiliations:** National Center of Hemostasis and Thrombosis, Department of Hematology and Transfusiology, Comenius University in Bratislava, Jessenius Faculty of Medicine in Martin and University Hospital in Martin, 03601 Martin, Slovakia; inkaskornova@gmail.com (I.S.); jan.stasko@uniba.sk (J.S.); jana.zolkova@gmail.com (J.Z.); juraj.sokol@uniba.sk (J.S.); palhol@gmail.com (P.H.); miroslava.dobrotova@gmail.com (M.D.); plamenova@pobox.sk (I.P.); hudecek@unm.sk (J.H.); simkovamonika@gmail.com (M.B.); alenka.stryckova@gmail.com (A.S.); peter.kubisz@uniba.sk (P.K.)

**Keywords:** von Willebrand factor, von Willebrand factor multimers, von Willebrand disease, electrophoresis, diagnostics, high-molecular weight multimers (HMW), intermediate-molecular weight multimers (IMW), low-molecular weight multimers (LMW)

## Abstract

von Willebrand disease (VWD) is reportedly the most common inherited bleeding disorder. This disorder develops as a result of defects and/or deficiency of the plasma protein von Willebrand factor (VWF). Laboratory testing for VWF-related disorders requires the assessment of both VWF level and VWF activity, the latter requiring multiple assays. As an additional step, an evaluation of VWF structural features by multimer analysis is useful in selective investigations. Multimer analysis is also important for the selection of a suitable VWF therapy preparation (desmopressin, VWF/FVIII concentrate, recombinant VWF) and the determination of the correct dose for the patient. Based on clinical and laboratory findings, including the analysis of VWF multimers, we classified our patients into individual types of VWD. Our study group included 58 patients. The study group consisted of 66% (38 patients) with VWD type 1, 5% (3 patients) with VWD type 2, 7% (4 patients) with VWD type 3, 5% (3 patients) with mixed type 1/2A VWD, and 17% (10 patients) comprising an unclassified group. In this article, we provide an overview of our practical experience using a new complementary method—the analysis of von Willebrand factor multimers with a semi-automatic analyzer Hydrasys 2 scan. We explain the principle, procedure, advantages, and pitfalls associated with the introduction of the VWF multimer analysis methodology into standard VWD diagnostics.

## 1. Introduction

Von Willebrand disease (VWD) reflects conditions caused by von Willebrand factor (VWF) deficiency and/or defects. Considered the most common inherited bleeding disorder, the true prevalence of VWD is unknown. Epidemiological studies estimate a value up to 1% of the population. Actual estimates may be higher in the general population as many people with a VWF mutation are asymptomatic or undiagnosed [1,2]. Similarly, a wide range of laboratory tests are used for its diagnosis, mainly because there is currently no standardized test available to unambiguously confirm or rule out the diagnosis of VWD [3,4].

VWF is a multimeric protein that performs two key functions in hemostasis. It is involved in the interaction between platelets and the subendothelium at the site of vascular damage, and in the interaction between platelets [5,6,7]. VWF also forms a complex with factor VIII, protecting it from proteolysis by activating protein C [8,9,10,11].

The adhesive activity of VWF depends on the presence of high-molecular weight (HMW) multimers. The analysis of VWF multimers, as well as determining the presence of HMW multimers and monitoring their quality and quantity, helps in the diagnosis of VWD, and can be used in the evaluation of VWD treatment and in monitoring responses to treatment [1,12].

The analysis of VWF multimers in practice using a semi-automatic device (Hydrasys 2 scan) was a challenge for us, not only owing to time constraints, but also because of the possibility of the quantitative evaluation of individual structures of VWF multimers. The aim was to provide a standardized complementary test in the diagnosis of VWD and, using the results of the VWF multimer analysis, to refine the laboratory diagnosis of von Willebrand disease and better classify patients with VWD into individual types and subtypes. Our goal was to move from a manual method to a standardized automated method, which increases the availability of examinations for a larger number of patients, and enables us to diagnose other patients and capture more evasive types of VWD.

The studies of Seidel, Bowyer, and Oliver et al. offer similar conclusions about the suitability of the method for inclusion into the routine VWD screening panel, owing to its time-efficiency, the reproducibility of its results, and its simple interpretability due to the possibility of the densitometric evaluation of individual VWF multimer structures [13,14,15].

Vasse et al. described the new test as a valuable tool for diagnosing hereditary or acquired VWD. It provides a clear picture of the distribution of VWF multimers, and its main advantage is that the result is available within one day. This paper presents clinical cases pointing to the importance of analyzing VWF multimers in therapy decisions [16].

Pikta et al. found the semi-automated system to be fast and sensitive, and useful not only for determining VWD types and subtypes, but also for monitoring therapy (e.g., after desmopressin administration) [17].

A study by Vangenechten and Gadisseur, comparing 40 healthy donors and 231 patients, found that the semi-automatic Hydrasys 2 scan method was suitable for clinical use, and was reliable and rapid. Although it cannot evaluate triplet structures, it is able to quantify the percentages of individual types of multimers. For this reason, this method may be applicable in other laboratories, in contrast to the current gold-standard manual method [18].

Boender et al. stated that the densitometric analysis of VWF multimers had excellent accuracy compared with visual multimer analysis, and may contribute to a better understanding of clinical features, such as the bleeding phenotype of VWD patients [19].

The objective of the study was to apply the semi-automatic Hydrasys 2 scan system to the analysis of VWF multimers and use it for the correct diagnosis in 58 patients with VWD.

## 2. Material and Methods

Our study enrolled 58 patients with VWD (38 female and 20 male) with a median age of 25.5 years (range 1–76 years). All patients were examined at the National Center for Hemostasis and Thrombosis at Martin University Hospital. Based on clinical manifestations or family history, they were assessed for VWD. Both the patients and the control group had blood drawn following the signing of informed consent.

The analysis of VWF multimers was performed via a semi-automatic Hydrasys 2 scan.

The semi-automatic Hydrasys 2 scan instrument (Sebia, Lisses, France), operates via the separation of proteins by means of innovative electrophoresis systems [13,20]. 

To analyze VWF, we used a multimers kit. The HYDRAGEL 5 von WILLEBRAND MULTIMERS (Sebia, Lisses, France) is a concentrated agarose gel intended for the separation of plasma proteins according to their molecular weight. The electrophoretic separation of VWF multimers was performed after sample treatment with an anionic detergent. When this anionic detergent is present in excess, proteins are converted into anionic detergent–protein complexes. In these complexes, the native conformation of proteins is disrupted, and they all assume the same conformation and the same negative charge per mass unit. When such anionic detergent–proteins are electrophoresed on a medium with appropriate sieving properties, such as HYDRAGEL 5 von WILLEBRAND MULTIMERS gel containing a high concentration of agarose, they separate according to their molecular weight. In HYDRAGEL 5 von WILLEBRAND MULTIMERS gels, VWF multimers with molecular weights between 500 and 20,000 kDa are separated and immunoprecipitated with a specific anti-VWF antiserum. The different bands within the gel are then visualized with a peroxidase-labeled antibody and a specific substrate. This assay is carried out in two stages: electrophoresis on agarose gel to separate the proteins contained in the plasma samples and immunofixation with an anti-VWF antiserum to visualize the different multimers. The semi-automated Hydrasys 2 instrument performs all the steps for obtaining gels that are ready for interpretation. This simple and fast technique gives a clear and easily interpretable picture.

### 2.1. Sample Preparation

Plasma samples were prepared from blood collected in tubes containing citrate as anticoagulant and directly analyzed or stored at −80 °C. Pooled plasma from healthy donors was used as a control. This group was selected for normal VWF levels and a negative history of bleeding events.

We diluted the patient sample with diluent to a ratio that depended on the VWF concentration (<20% VWF: dilution 1/4; 20–150% VWF: dilution 1/6; 150–300% VWF: dilution 1/10; >300% VWF: dilution 1/20).

### 2.2. Electrophoresis

**Figure 1:**
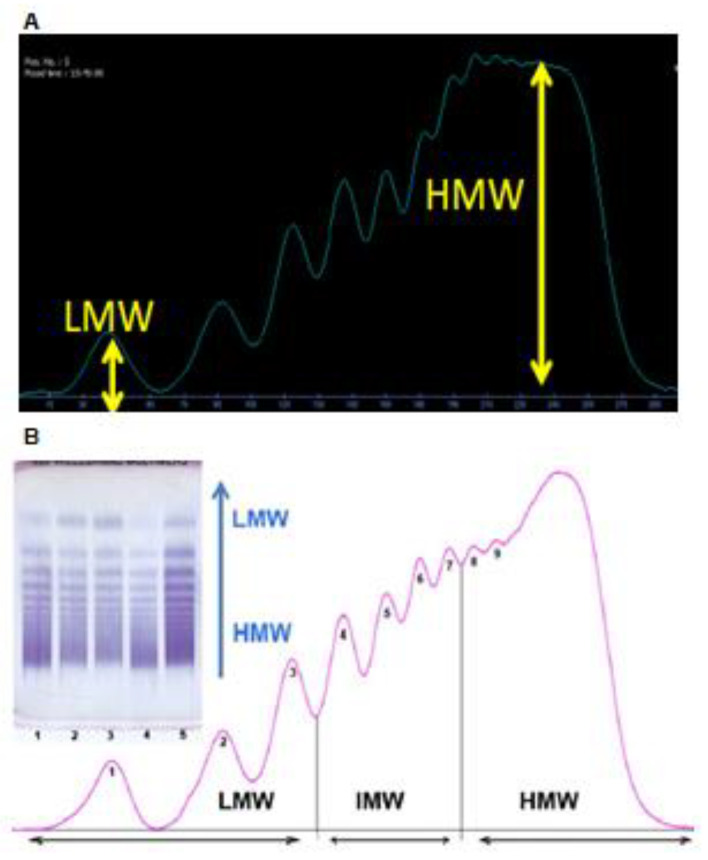
Densitometric representation of VWF multimers. (**A**) Peak intensity directly correlates with VWF multimer concentration. (**B**) Densitometric representation of peaks from left to right, with peaks 1–3 being LMW, peaks 4–7 being IMW, and all other peaks forming HMW (own data).

The diluted sample and control plasma were placed in the wells of a 2% prepared separation gel. Only one concentration of agarose was used in this method. We turned commenced electrophoresis at 90 V and 10 mA with a duration setting of 110 min and plate cooling at 15 °C. Electrophoretic separation was performed according to the molecular weight in the neutral buffer containing anionic detergent. The low-molecular weight (LMW) multimers are located on the top of the gel and at the left side of the *x*-axis of the densitogram. Correspondingly, HMW multimers are located on the bottom of the gel and at the right side of the *x*-axis of the densitogram, and intermediate-molecular weight multimers (IMW) multimers are located in between. Although there is no consensus on the definition of the areas comprising LMW, IMW, and HMW multimers, for convenience in interpreting the results, the multimer bands of this quartet of healthy subjects were classified as follows: 1–3 left to right peaks in the densitogram would represent LMW multimers, peaks 4–7 would represent IMW multimers, and peaks 8 and onwards would represent the group of HMW multimers (Figure 1) [17].

### 2.3. Immunofixation

**Figure 2:**
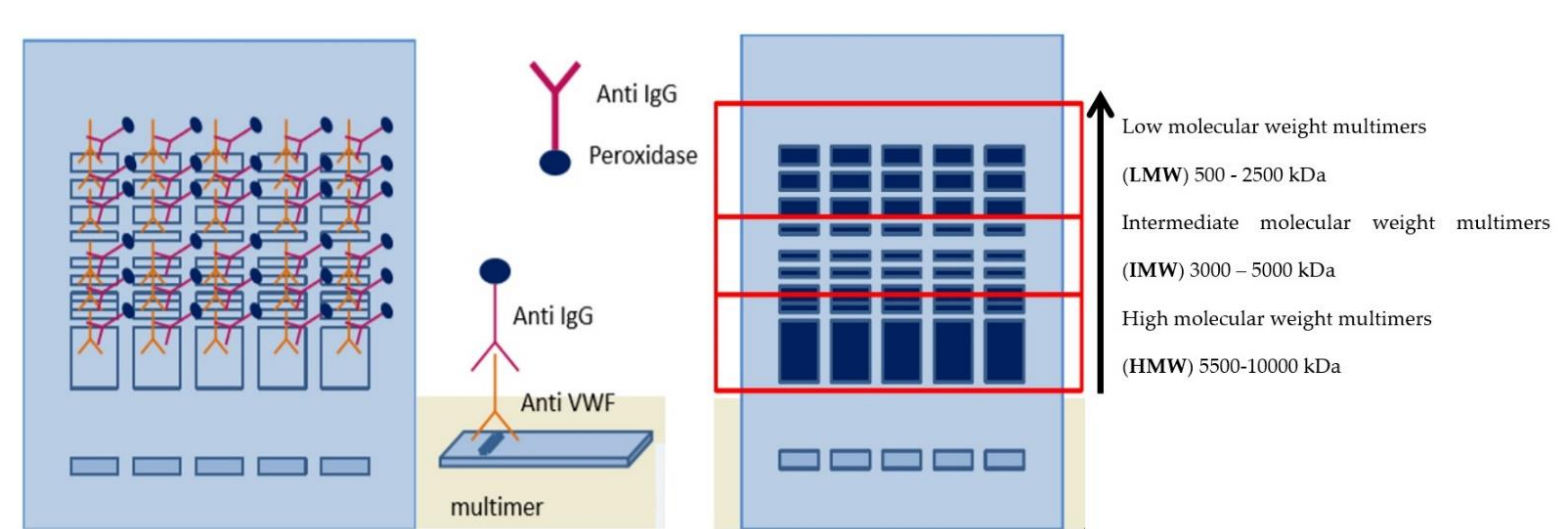
Principle of the determination and visualization of VWF multimers via the semi-automatic method of Hydrasys 2 scanning. Incubation of the first immunofixation of VWF multimers in a gel with a polyclonal anti-VWF-IgG antibody, and incubation of the second immunofixation with peroxidase-conjugated anti-IgG antibody. The gel was subsequently incubated in peroxidase and chromogen substrate (TTF1/TTF2), then dried and prepared for interpretation (own data).

The first direct immunofixation of VWF multimers in a gel involved a polyclonal, anti-VWF-IgG antibody with an incubation duration of one hour. Excess protein was eliminated via elution. The incubation of the second immunofixation with peroxidase-conjugated anti-IgG antibody lasted 30 min (Figure 2). Excess proteins were eliminated by elution.

### 2.4. Visualization

The gel was subsequently incubated for 10 min in peroxidase and chromogen substrate (TTF1/TTF2). Excess reagent was removed, and the gel was dried and prepared for interpretation (Figure 2). Gels containing the analyzed sample were scanned with a Hydrasys 2 and densitometrically evaluated and quantified relative to the control (Figure 1). We have evaluated the densitometric representation of the peaks according to the manufacturer’s recommendations from left to right, with peaks 1–3 being LMW, 4–7 being IMW, and all other peaks being HMW (Figure 1) [21,22]. We used the Phoresis software (Version: 8.63P11, Sebia, Lisses, France) to quantify the LMW, IMW, and HMW multimers, and the intensity of the peaks directly correlated with the concentration of VWF multimers [23].

In the laboratory, we performed screening tests comprising a complete blood count, including the platelet count (Plt) (Beckman Coulter, Inc., Brea, CA, USA) and bleeding time (BT) (Duke method, physiological time interval 2–5 min); analysis of platelet function by PFA analyzer (normal range for collagen/ADP 62–100 s and collagen/EPI 82–150 s; Dade Behring Inc., Brookfield, CT, USA); activated partial thromboplastin time (APTT; Werfen/Instrumentation Laboratory, Bedford, MA, USA); specific tests including the determination of factor VIII plasma activity (FVIII: C; Werfen/Instrumentation Laboratory, Bedford, MA, USA) (one-step method, normal range (NR): 60–150%); and VWF collagen binding activity (VWF: CBA; Werfen/Instrumentation Laboratory, Bedford, MA, USA) (chemiluminescent method, type III collagen, NR: 50–150%). VWF antigen (VWF: Ag; Siemens Healthcare Diagnostics, Erlangen, Germany) (ELISA, NR: 60–150%); VWF activity (VWF: Ac; Siemens Healthcare Diagnostics, Erlangen, Germany) (turbidimetric method—recombinant GPIb is added to agglutination and turbidity is measured in microparticles coated with a mouse anti-GPIb antibody directed against a functional VWF epitope with a GPIb binding site, NR: 50–140%).

In the diagnosis, we used additional tests in some patients, mainly to determine the qualitative subtype of VWD. These included the ristocetin-induced platelet aggregation (RIPA) test; VWF binding to FVIII (VWF: FVIII) (ELISA, NR: 20–100%); VWF propeptide (VWF: pp) (ELISA, NR: 0.6–2.2 IU/mL); and genotype tests. The genotype analysis helped us to distinguish between severe type 1 VWD and type 3 VWD, and served to confirm the mixed phenotype 1/2A of VWD [24].

## 3. Results

The rational diagnostic strategy for patients with VWD is based on phenotypic analysis and, in specific cases, genotypic analysis.

In our study, we analyzed the normal plasma of 10 blood donors aged 19 to 59 (median of 44 years). The normal ranges of VWF multimers that we obtained were evaluated quantitatively using the Phoresis program. The normal ranges in our cohort were 12–24% for LMW, 25–35% for IMW, and 41–70% for HMW, in agreement with previous published studies [18,25] and the manufacturer’s recommendations. The individual types of VWF multimers in patients were quantitatively determined in comparison with the control.

In the donors, the values were as follows: FVIII—Ac 80–130%, median 95%; VWF—Ag 70–120%, median 89%; VWF—Ac 60–130%, median 90%; VWF—CBA 70–120%, median 85%.

The results for patients diagnosed with VWD type 1 were as follows: FVIII—C 0.9–195%, median 72%, VWF—Ag 0.5–90%, median 90%; VWF—Ac 0.8–70%, median 39%; VWF—CBA 7–105%, median 58%. The following are the blood groups of the patients with VWD type 1: O blood group: *n* = 15, A blood group: *n* = 9, B blood group: *n* = 5, AB blood group *n* = 2; 7 patients had a non-identifiable blood group.

The results of patients diagnosed with VWD type 2 were as follows: FVIII—C 34–49%, median of 39%; VWF—Ag 15–108%, median of 46%; VWF—Ac 7.6–129%, median of 26%—VWF: CBA 6–73%, median of 28%. One patient with VWD 2 was O blood group and two patients’ blood groups were non-identifiable.

The results of patients diagnosed with VWD type 3 were as follows: FVIII—C 5–16%, median of 10.5%; VWF—Ag 0.5–6%, median of 3.7%; VWF—Ac 0.5–19%, median of 4.05%; VWF—CBA 6–27%, median of 16.5%. The blood groups of patients with VWD type 3: O blood group: *n* = 1, B blood group: *n* = 1, AB blood group *n* = 1, and one patient had a non-identifiable blood group.

The results of patients diagnosed with mixed type VWD 1/2A were as follows: FVIII—C 49–67%, median of 62%; VWF—Ag 11–41%, median of 32%; VWF—Ac 15–29%, median of 18%; VWF—CBA 6.4–15%, median of 11%. The blood groups of patients with mixed type VWD 1/2A: B blood group: *n* = 1, AB blood group *n* = 2.

The results of patients with unclassified VWD were as follows: FVIII—C 59–150%, median of 96%; VWF—Ag 32–124%, median of 61.5%; VWF—Ac 10–120%, median of 49%; VWF—CBA 11–97%, median of 71%. The blood groups of patients unclassified VWD were: O blood group: *n* = 4, A blood group: *n* = 1, and five patients had non-identifiable blood groups.

**Figure 3:**
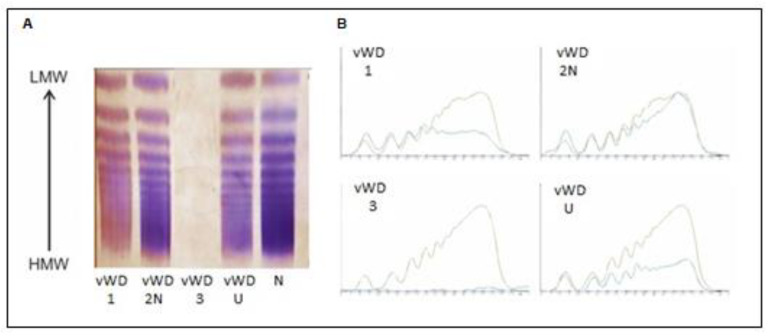
Analysis of VWF multimers of patients with VWD type 1 (sample number 1 in Table 1), VWD type 2N (sample number 2 in Table 1), VWD type 3 (sample number 3 in Table 1), and VWD U (unclassified) (sample number 4 in Table 1); (**A**) semi-automatic Hydrasys 2 scan and (**B**) densitometric evaluation.

Based on clinical manifestations, the test results, and the analysis of VWF multimers, we classified the patients into VWD types. The study group comprised 66% (38 patients) with VWD type 1, 5% (3 patients) with VWD type 2, 7% (4 patients) with VWD type 3, 5% (3 patients) with mixed type 1/2A VWD, and 17% (10 patients) formed an unclassified group. We analyzed the VWF multimers of samples VWD 1, VWD 2N, VWD 3, and VWD U (unclassified) on the Hydrasys 2 semi-automatic instrument, performing densitometric evaluations as shown Figure 3. The results for patients with VWD type 1, VWD type 2N, VWD type 3, and VWD U (unclassified) are shown Table 1. The results for the VWF—Ac, VWF—Ag, CBA, and VWF multimers are shown Table 2.

## 4. Discussion

**Figure 4:**
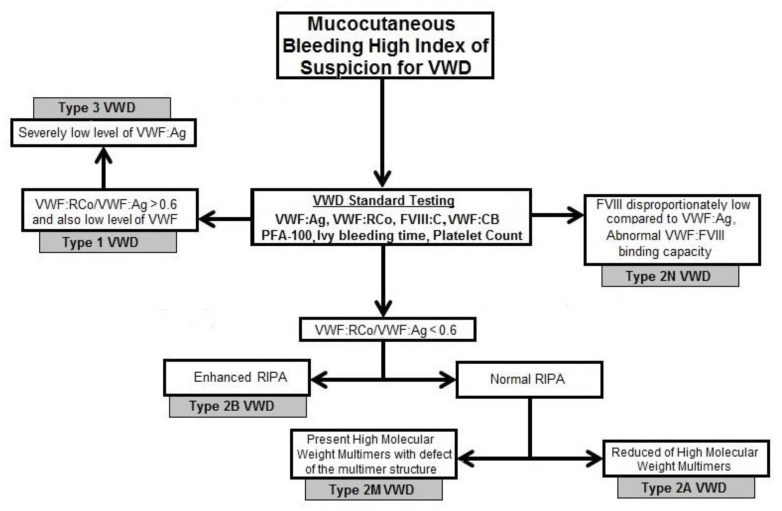
Algorithm for the diagnosis and classification of von Willebrand disease.

Based on the clinical manifestations and diagnostic results, we classified patients into individual types of VWD. The review article describes the algorithm used in the diagnosis of VWD. This diagnostic algorithm was also used in our study (Figure 4) [26].

In our study group, 38 patients (the largest group) had VWD type 1, which is consistent with the data described by Favaloro et al. [27], who stated that, in developed countries, VWD type 1 usually comprises more than 60% of the identified cases of VWD. This group consists of people who have mild bleeding symptoms and either normal or low VWF levels. At the same time, there are some who have low levels of VWF, but no signs of bleeding. In addition, VWF levels in individuals also differ on a genetic basis, with the most common effect being found in the ABO blood group—individuals with blood group O have about 10–20% lower plasma VWF levels [28,29].

The current international recommendations suggest that patients with VWD type 1 should not be diagnosed unless their VWF levels are below 0.30 IU/mL [30]. This is because mutations in the VWF gene associated with VWD type 1 usually only occur in these cases [31,32].

VWD type 1 is the mildest form of VWD in terms of bleeding symptoms. However, with more marked reductions in VWF activity, bleeding symptoms may be exacerbated, signaling severe VWD type 1.

In our cohort, the patients had a mild form of VWD type 1; their platelet counts were normal, and the times required for determination of their functional platelet counts with the PFA analyzer were extended. The ratio of VWF activity to antigen was >0.7 in the samples. All VWF multimers were detected by quantitative analysis. A comparison of the VWF multimers obtained from patients with those in the control plasma by quantitative densitometric evaluation confirmed the lower concentration of the former.

We classified three patients as severe VWD type 1 and two patients as moderate VWD type 1. The patients had normal platelet counts, but their bleeding times were prolonged, their functional platelet assays via the PFA analyzer were extended, and their VWF activity ranges were 0.008–0.22 IU/mL. We performed the VWF—pp test on three patients, deriving < 0.6 IU/mL in two cases (NR: 0.6–2.2 IU/mL), and the mutation for severe VWD type 1 was genetically confirmed in one case. All types of VWF multimers were significantly reduced or absent in all samples. The patients reported epistaxis, bleeding gums, hematomas in the body, recurrent lower GIT bleeding, and hemorrhagic conditions.

Because our cohort was relatively small, only three patients were classified with VWD type 2. In one sample, the platelet count and closure time (CT) ranges for PFA were normal, and VWF—Ac, VWF—Ag, and CBA were also normal. All VWF multimers were assessed as present by the quantitative analysis of multimers. Owing to the reduced range of FVIII plasma activity, an assay of VWF to FVIII binding capacity was performed to confirm the presence of VWD type 2N. The VWF—FVIII test result confirmed the presence of VWD type 2N.

In two samples, the platelet counts were normal; the CT time on the PFA analyzer was prolonged; VWF—Ac, VWF—Ag, CBA, and FVIII were reduced; the VWF/VWF—Ag ratio was <0.57 and the CBA/VWF—Ag ration was <0.61, suggesting VWD type 2. The analysis of VWF multimers by quantitative densitometric evaluation showed reductions in both HMW and IMW compared with the control pool. The samples were analyzed for ristocetin-induced platelet aggregation (RIPA) at low ristocetin concentrations, without confirming hyperagregability. The results refer to both VWD type 2A samples.

Global data on the prevalence of VWD type 3 vary significantly. VWD type 3 is considered a rare type in developed countries, usually representing <5% of all VWD cases, with an incidence of 1–2 million across the population. Surprisingly, Slovakia has one of the highest rates of VWD type 3 incidence across the world [26].

Our group contained four patients with VWD type 3. In the samples, the platelet count was normal; CT on PFA was significantly prolonged, with the ranges of VWF—Ac, VWF—Ag, CBA, and FVIII showing significant reductions. VWF multimers and their quantifiable structures were not detectable in the samples.

Our cohort contained three patients with mixed type VWD 1/2A. In the samples, the platelet count ranges were normal, the CT measured on PFA was prolonged, the activity and the VWF antigen were reduced proportionally, and the CBA range was also reduced. Quantitative analyses of VWF multimers confirmed the deficit of IMW and HMW multimers. In all three samples, a mutation was genetically detected that caused a mixed VWD type 1/2A phenotype (subtype IIE) in some individuals. In these patients, we made a laboratory finding of type 1 VWD, but with a multimer profile typical for type 2A/IIE VWD.

The last subgroup of patients consisted of 10 patients with unclassified VWD. In the monitored samples, the platelet count was normal, there was decreased VWF activity and antigen ranges, the VWF/VWF—Ag ratio was >0.7, and FVIII and CBA were normal. In addition, the VWF multimers had reduced IMW and HMW ranges compared with the control pool.

The patients currently enrolled in our study as unclassified VWD should be repeatedly examined over time with additional tests, including genotypic analyses.

Favaloro et al. [33] also cited a group of VWD patients not further classified in Australia in their review of VWD patients. Batlle et al. described the results of a multicenter study in Spain with a group of 556 patients with VWD from 330 families. All patients with VWD were subjected to next-generation sequencing (NGS) of their entire VWF coding gene compared with the phenotype, and the diagnosis of VWD was repeated. In total, 238 different VWF mutations were found. The study also included a group of undefined patients, or those without a VWD phenotype that was not further classified [34].

In a study by Flood et al., comprising all patients with previously diagnosed VWD in the national Zimmerman program in the United States, the authors concluded that the VWF—Ac levels were normalized in some patients. The authors of this study explain this via the fact that VWF levels increase with age, such that a relatively mild VWF deficiency may normalize over time. Furthermore, in elderly patients, concomitant inflammatory conditions may occur, leading to an increase in and normalization of VWF levels. A third possibility is that some patients were diagnosed with VWD based on one laboratory-measured range that was lower than the reference interval in the normal population [35]. In the comparison of the representation of individual types and subtypes of VWD, our data did not differ from those published elsewhere [36,37].

Based on our experience with the Hydragel 5 von Willebrand Factor Multimer kit for VWF multimer analysis on a semi-automated Hydrasys instrument, we can say that this method has its advantages and disadvantages. The advantage is that it is a standardized method offering reproducible results, the solutions and gels are already prepared, and the results are available on the day of analysis.

The visual evaluation as part of the manual method can be influenced by subjective attitudes. Conversely, densitometric and quantitative analyses of VWF multimers via Hydrasys 2 scanning can provide information on the presence (normal distribution or detection with reduced optical density) or absence of VWF multimers. Densitometry improved the interpretation of VWF multimers that had undergone small structural changes that were not evaluable via visual inspection in the manual method.

The main advantages of the analysis performed with the Hydrasys semi-automatic analyzer compared with manual analysis are that the results are available on the day of the analysis, making them immediately helpful for further management of the patient’s treatment. A limitation of the method using the Hydrasys analyzer is the impossibility of determining in the laboratory the VWD type 2A subtypes (IIA, IIE, IIC, IID) at the level of the triplet structure of individual multimers [18].

The disadvantage of the Hydrasys 2 method is that it does not provide a representation of the triplet structure, which is essential when determining the 2M type of VWD; however, this method is crucial for VWD type 2A classification (subtypes IIA, IID, IIE, and IIC) [20,22]. The Hydrasys 2 method is also very important in the diagnosis and treatment of VWD type 2B, which has abnormal HMW multimers (in about two-thirds of patients with VWD type 2B). For patients in the VWD type 2B subgroup with normal HMW multimers (about one-third of patients with VWD type 2B), desmopressin or platelet concentrate may be used in the treatment of bleeding. In contrast, these products are contraindicated in VWD type 2B patients with abnormal HMW multimers in the treatment of bleeding. In this subgroup of patients with VWD type 2B, only VWF/FVIII concentrates can be used in the treatment, similar to VWD type 2A, 2N, severe VWD type 1 (VWD type 1C (Vicenza)), and VWD type 3 [38].

In the case of VWD type 2N, owing to the presence of atypical mutations, in addition to the erroneous binding of VWF to FVIII, slight quantitative deficiencies and abnormal multimers may occur [39].

The VWF manual multimer analysis method used previously can be used to carry out triplet multimer analysis, but the method is non-standardized and has lower reproducibility, with gels and solutions needing to be prepared in each assay. The method is also very time-consuming and technically demanding. Examination via both methods requires the processing and evaluation of samples in a central laboratory with expertise and specialized instrumentation.

## 5. Implications for Clinical Practice

The structure of VWF multimers is a complex result of VWF metabolism, occurring via synthesis, post-translational modification, secretion, proteolysis, consumption, and clearance. Changes in individual processes lead to characteristic changes of VWF multimers. Taking this into account, the analysis of VWF multimers can be used to further characterize the VWD type (in particular, qualitative disorders) and to identify any disorders in the VWF metabolization process.

The analysis of VWF multimers can be performed in patients to determine the type of VWD and the pathomechanism related to changes in any process during VWF metabolism, in order to evaluate the presence of VWF–HMW multimers in VWF coagulation factor VIII concentrates and VWF concentrate, as well as for monitoring the response to treatment. Our goal is to analyze VWF multimers in order to specify the subtypes of VWD type 2, which will improve the treatment of these patients.

Based on data from the available literature, as well as our own experience, multimer analysis should be indicated if there is suspicion of a qualitative VWF defect (2A, 2B), as well as a quantitative deficiency (VWD type 3), acquired von Willebrand’s syndrome (AVWs) or thrombotic thrombocytopenic purpura (TTP).

The analysis of VWF multimers is important not only in the diagnosis of VWD, but also for the evaluation of the treatment of this most common congenital bleeding disorder. The quantitative and qualitative evaluation of VWF multimers is effective and useful for the screening, detection, and classification of VWD subtypes, and makes this method diagnostically and clinically relevant.

## Figures and Tables

**Table 1:** Results for patients with VWD type 1, VWD type 2N, VWD type 3, and VWD U (unclassified).

Sample No.	VWF—Ac (%) NR: 50–140%	VWF—Ag (%) NR: 60–150%	VWF—Ac/VWF—Ag	FVIII—C (%) NR: 60–150%	CBA (%) NR: 50–150%	CBA/VWF—Ag	Multimers (%) LMW (NR: 12–24%) IMW (NR: 25–35%) HMW (NR: 41–70%)	VWD Type
1	34	46	0.74	101	105	2.28	LMW 28% IMW 25% HMW 26%	VWD type 1
2	129	108	1.19	39	73	0.68	LMW 20% IMW 25% HMW 45%	VWD type 2N
3	7	0.5	uncalculable	5	6	uncalculable	LMW 3.3% IMW 1% HMW 3%	VWD type 3
4	26	38	0.68	92	71	1.87	LMW 17.4% IMW 17.5% HMW 27%	VWD unclassified

VWF: von Willebrand factor; VWF—Ac: VWF activity; VWF—Ag: VWF antigen, FVIII—C: factor VIII plasma activity; CBA: collagen binding assay; NR: normal range; VWD: von Willebrand disease; N: normal control; U: (unclassified); sample no.: sample number.

**Table 2:** The laboratory results and type VWD of all patients.

Sample No.	VWF—Ac % NR: 50–140	VWF—Ag % NR: 60–150	VWF—Ac/ VWF—Ag	FVIII % NR: 60–150	CBA % NR: 50–150	CBA/VWF—Ag	Multimers (%) LMW (NR: 12–24%) IMW (NR: 25–35%) HMW (NR: 41–70%)	VWD
1	24	32	0.75	71	31	0.97	LMW 10% IMW 15% HMW 24%	VWD type 1
2	0.5	3	0.17	9	<0.5	uncalculable	LMW 4% IMW 2% HMW 0%	VWD type 3
3	7	0.5	uncalculable	5	6	uncalculable	LMW 3.3% IMW 1% HMW 3%	VWD type 3
4	26	38	0.68	92	71	1.87	LMW 17.4% IMW 17.5% HMW 27%	VWD unclassified
5	34	46	0.74	101	105	2.28	LMW 28% IMW 25% HMW 26%	VWD type 1
6	129	108	1.19	39	73	0.68	LMW 20% IMW 25% HMW 45%	VWD type 2N
7	18	32	0.56	67	15	0.47	LMW 13% IMW 10% HMW 14%	VWD type 1/2A
8	29	41	0.71	62	11	0.27	LMW 12% IMW 7% HMW 8%	VWD type 1/2A
9	45	54	0.83	80	65	1.20	LMW 7% IMW 4% HMW 5%	VWD unclassified
10	50	55	0.91	59	59	1.07	LMW 6% IMW 6.8% HMW 6%	VWD unclassified
11	86	110	0.78	150	71	0.65	LMW 10% IMW 17% HMW 29%	VWD unclassified
12	58	56	1.04	69	71	1.27	LMW 10% IMW 17% HMW 29%	VWD type 1
13	25	38	0.66	195	31	0.82	LMW 34% IMW 32% HMW 45%	VWD type 1
14	44	50	0.88	58	59	1.18	LMW 12% IMW 17% HMW 35%	VWD type 1
16	69	64	1.08	120	76	1.19	LMW 13% IMW 19% HMW 30%	VWD type 1
17	64	83	0.77	90	72	0.87	LMW 25% IMW 24% HMW 36%	VWD type 1
18	65	90	0.72	90	82	0.91	LMW 12% IMW 16% HMW 26%	VWD type 1
20	47	116	0.41	104	73	0.63	LMW 33% IMW 40% HMW 41%	VWD unclassified
24	120	124	0.97	100	97	0.78	LMW 35% IMW 43% HMW 65%	VWD unclassified
25	47	55	0.85	44	81	1.47	LMW 15% IMW 19% HMW 38%	VWD type 1
26	1.1	4.4	0.25	16	<0.5	uncalculable	LMW 3.2% IMW 1.5% HMW 0%	VWD type 3
27	2	0.5	uncalculable	3.5	7	uncalculable	LMW 1.7% IMW 0.1% HMW 1.9%	VWD type 1
28	63	68	0.93	91	73	1.07	LMW 25% IMW 8% HMW 16%	VWD type 1
29	67	65	1.03	122	63	0.97	LMW 23% IMW 20% HMW 37%	VWD type 1
30	70	80	0.88	50	72	0.90	LMW 17% IMW 12% HMW 21%	VWD type 1
38	55	58	0.95	92	52	0.90	LMW 37% IMW 28% HMW 35%	VWD unclassified
39	27	33	0.82	60	10	0.30	LMW 22% IMW 15% HMW 23%	VWD type 1
42	22	33	0.67	29	20	0.61	LMW 9% IMW 1% HMW 6%	VWD type 1
43	15	25	0.60	45	30	1.20	LMW 11% IMW 3% HMW 13%	VWD type 1
44	39	55	0.71	136	72	1.31	LMW 25% IMW 30% HMW 42%	VWD type 1
48	60	66	0.91	90	70	1.06	LMW 17% IMW 17% HMW 30%	VWD type 1
49	0.8	0.5	1.60	0.9	<0.5	uncalculable	LMW 9% IMW 1% HMW 7%	VWD type 1
50	7.6	15	0.51	34	6	0.40	LMW 14% IMW 2.5% HMW 11%	VWD type 2A
51	44	50	0.88	88	47	0.94	LMW 12% IMW 9% HMW 23%	VWD type 1
52	15	11	1.36	49	6.4	0.58	LMW 17.3% IMW 1.1% HMW 5.8%	VWD type 1/2A
53	26	46	0.57	49	28	0.61	LMW 34% IMW 14% HMW 8%	VWD type 2A
54	10	32	0.31	101	11	0.34	LMW 39% IMW 41% HMW 61%	VWD unclassified
55	36	38	0.95	59	35	0.92	LMW 17% IMW 8% HMW 17%	VWD type 1
56	23	32	0.72	63	37	1.16	LMW 15% IMW 12% HMW 31%	VWD type 1
57	48	65	0.74	82	73	1.12	LMW 16% IMW 14% HMW 33%	VWD unclassified
58	72	77	0.94	124	73	0.95	LMW 19% IMW 15% HMW 34%	VWD unclassified
59	19	6	uncalculable	12	27	uncalculable	LMW 14% IMW 4% HMW 5%	VWD type 3
60	34	36	0.94	69	35	0.97	LMW 11% IMW 11% HMW 27%	VWD type 1
61	16	25	0.64	21	20	0.80	LMW 22% IMW 14% HMW 19%	VWD type 1
62	56	54	1.04	100	81	1.50	LMW 20% IMW 15% HMW 37%	VWD type 1
63	36	30	1.20	123	41	1.37	LMW 2% IMW 3% HMW 35%	VWD type 1
64	39	41	0.95	80	57	1.39	LMW 15% IMW 8% HMW 29%	VWD type 1
65	30	34	0.88	59	52	1.53	LMW 13% IMW 10% HMW 29%	VWD type 1
66	22	25	0.88	72	60	2.40	LMW 12% IMW 9% HMW 24%	VWD type 1
67	44	45	0.98	111	35	0.78	LMW 11% IMW 11% HMW 22%	VWD type 1
68	35	30	1.17	81	59	1.97	LMW 12% IMW 9% HMW 13%	VWD type 1
69	61	58	1.05	99	58	1.00	LMW 14% IMW 15% HMW 24%	VWD type 1
70	15	28	0.54	70	14	0.50	LMW 10% IMW 7% HMW 13%	VWD type 1
71	15	24	0.63	58	15	0.63	LMW 8% IMW 7% HMW 15%	VWD type 1
72	45	63	0.71	72	65	1.03	LMW 18% IMW 14% HMW 21%	VWD type 1
73	42	48	0.88	64	57	1.19	LMW 17% IMW 11% HMW 19%	VWD type 1
74	62	53	1.17	97	78	1.47	LMW 10% IMW 11% HMW 24%	VWD type 1
75	48	69	0.70	111	59	0.86	LMW 12% IMW 10% HMW 30%	VWD type 1

## Data Availability

All the data are available from the Corresponding Author (tomas.simurda@uniba.sk) upon reasonable request.
